# Which one has a better obstetric and perinatal outcome in singleton pregnancy, IVF/ICSI or FET?: a systematic review and meta-analysis

**DOI:** 10.1186/s12958-016-0188-3

**Published:** 2016-08-30

**Authors:** J. Zhao, B. Xu, Q. Zhang, Y. P. Li

**Affiliations:** Department of Reproductive Medicine, Xiangya Hospital, Central South University, 87 Xiangya Road, Changsha City, 410008 Hunan Province People’s Republic of China

**Keywords:** In vitro fertilization (IVF), Intracytoplasmic sperm injection (ICSI), Perinatal outcomes, Obstetric outcomes, Frozen embryo transfer (FET)

## Abstract

**Background:**

The present study aims to compare which one has a better obstetric and perinatal outcome in singleton pregnancy, frozen embryo transfer (FET) or. in vitro fertilization treatment/intracytoplasmic sperm injection (IVF/ICSI)?

**Methods:**

MEDLINE, Google Scholar and the Cochrane Library were searched for the obstetric and perinatal outcomes in singleton pregnancy after assisted reproductive technology (ART) from inception until July 2016. Clinical trials, which compared obstetric/perinatal outcomes in singleton pregnancy after FET and IVF/ICSI-ET, were included. The primary outcome was low birth weight, preterm birth, perinatal mortality, still birth, and cesarean section.

**Results:**

Thirteen cohort studies with 126,911 women were included, of which 12, 11, 6, 6, 5 studies were used to analyze low birth weight, preterm birth, perinatal mortality, still birth, and cesarean section, respectively. IVF/ICSI is associated with a high risk of preterm birth (OR = 1.14, 95 % CI: 1.02, 1.28) and low birth rate (OR = 1.48, 95 % CI: 1.37, 1.60). There was no significant difference in the risk of the still birth (OR = 1.01, 95 % CI: 0.76, 1.35) and perinatal mortality (OR = 1.11, 95 % CI: 0.85, 1.46) between FET and IVF/ICSI. Singleton pregnancy after FET was associated with higher cesarean section rate compared with IVF/ICSI (OR = 0.85, 95 % CI: 0.80, 0.91).

**Conclusions:**

Singleton pregnancy after FET seems to have a better perinatal outcome compared with that after IVF/ICSI. Further randomized controlled trials which adjust for a variety of meaningful confounders are needed in order to draw sound conclusions.

**Electronic supplementary material:**

The online version of this article (doi:10.1186/s12958-016-0188-3) contains supplementary material, which is available to authorized users.

## Background

Controlled ovarian stimulation (COS) was reported to have enhanced the incidence of ovarian hyperstimulation syndrome (OHSS). Besides, a number of epidemiological and population-based studies have suggested that COS followed by fresh transfer may result in increased risk of perinatal outcomes in pregnancies [1–6]. Compared with the in vitro fertilization/ intracytoplasmic sperm injection (IVF/ICSI) cycles which need complex stimulation protocols to gain multiple follicular growth, FET are simpler and safer, with only one aim of preparing a receptive endometrium. The superfluous viable embryos were cryopreserved, and would be transferred in the succeeding cycles. The accumulative success rate could be improved after one ovarian stimulation and retrieval cycle. Therefore, along with the refinement of laboratory techniques, the proportion of FET has increased [7] dramatically since the first baby after the frozen-thawed embryo transfer (FET) cycle was born in 1984 [8, 9].

With the wide use of FET, there were concerns about the negative effect of cryopreservation on the health of children born. A number of observational studies [10–12] suggested that both obstetric and perinatal outcomes after FET are similar to those after IVF/ICSI cycles, whereas other investigators indicated that they were even better than those of fresh IVF/ICSI cycles [13, 14]. These observational studies suggested that there was no significant difference in pivotal perinatal outcome because of the flaws in design of the study. Regrettably, there was no randomized controlled trial which compared obstetric and perinatal outcomes after FET cycles with those after fresh IVF/ICSI cycles.

Because these obstetric and perinatal outcomes were mostly resulting from multiple gestations, owing to multiple ETs, it is reasonable to compare the obstetric and perinatal outcomes in single embryo transfer (SET) cycles as they have the advantage of reducing multiple pregnancy rates. An early meta-analysis and systematic review [1] compared the obstetric and perinatal outcomes after FET with singleton pregnancy with those after fresh IVF cycles, and concluded that pregnancies after FET cycles may have a better result with regard to the obstetric and perinatal outcomes. Although ten outcome measurements were evaluated, the studies and samples were relatively small.

Hence, it is urgent to perform a meta-analysis with larger samples to compare the obstetric and perinatal outcomes in singletons pregnancy after FET and IVF/ICSI. The present study includes additional three studies and aims to evaluate which one has a better perinatal outcome of singleton pregnancy, IVF/ICSI or FET.

## Methods

### Literature identification

MEDLINE, Google Scholar and the Cochrane Library were searched from inception until July 2015. The keywords were used to search relative studies: one including terms on obstetric or perinatal outcomes (obstetric outcome, obstetric complication, perinatal outcome, perinatal complication), the other one about reproductive techniques (in vitro fertilization, intracytoplasmic sperm injection, frozen embryo transfer, IVF, ICSI, FET). We combined these subsets with “AND” to get a number of publications associated with our analysis. Papers published in non-English were excluded. The papers were reviewed by two investigators independently, and a third author was needed when there was a disagreement.

### Study selection and data extraction

Studies which compared the obstetric or perinatal outcomes in singleton pregnancy after IVF/ICSI vs. FET were selected. The primary outcome of interest was preterm birth and/or low birth weight and/or still birth and/or perinatal mortality and/or cesarean section. For studies to be eligible, 2 × 2 tables were used for outcome data extraction. We also recorded the treatment type, number of cycles and number of obstetric/perinatal complications. If necessary, we would contact research author to clarify the data. The quality of the observational studies was assessed by Newcastle-Ottawa Quality Assessment Scales [15]. The quality of the publications included was evaluated by two reviewers, and a third reviewer was needed when there was any disagreement about inclusion.

### Statistical analysis

Meta-analysis was attempted wherever appropriate. The data of each study was extracted in 2 × 2 tables. Odds Risks (ORs) and risk differences with 95 % Confidence Intervals (CI) was used to describe the dichotomous outcomes of each study. Forest plots were used to evaluate the heterogeneity of the exposure effects graphically and l^2^ was implied to assess the heterogeneity between studies. A sensitivity analysis was performed by altering the fixed-to-random effect analysis in the event of moderate heterogeneity (l^2^ > 50 %). A *P*-value of ≤ 0.10 rather than the conventional level of ≤ 0.05 was used to determine statistical significance because the *X*^2^ test for heterogeneity has low power in a meta-analysis especially the study had a small sample size. RevMan 5.0 (Cochrane Collaboration, Oxford, UK) was implied for statistical analyses.

## Results

### Studies selection and characteristics

The search strategy yielded 823 records. 783 papers were not found relevant after review of the titles and abstracts. Of the 40 remaining publications, 24 were excluded with all kinds of reasons (no relative data available *n* = 21; sample size were not mentioned *n* = 2; methodological concern *n* = 1). One study was excluded since its results were duplicated with another paper that has been included in our study. An additional three papers were excluded because a 2 × 2 table would not be extracted from the result (Additional file 1: Figure S1).

Thirteen eligible studies, which reported obstetric or/and perinatal outcomes after IVF/ICSI vs. FET cycles, with 126,911 infertile women were included in the present review. The study characteristics are depicted in Table 1. In the included studies, verification or slow freezing techniques were implied for embryos were frozen on day 2/3 (cleavage stage) or day 5/6 (blastocyst stage). Natural/artificial/stimulated protocols for preparing endometrium were used for frozen embryo transfer.

**Table 1:** Characteristics of studies included in the meta-analysis

Study		Area/duration	Type of study	Population	Embryo transferred		Freezing techniques	FET protocol	Measurements assessed
					Fresh ET	FET			
1994	Wada	UK 1985–1991	Retro	IVF vs. FET	D2/D3 embryos	Cleavage embryos/blastocysts	Slow freezing	Natural/HRT cycles	Preterm birth LBW Still birth
2005	Wang	Australia 1996–2000	Retro	IVF/ICSI/GIFT vs. FET	Not mentioned	Not mentioned	Not mentioned	Not mentioned	Preterm birth LBW
2008	Belva	Belgium 1983–2006	Retro	IVF/ICSI vs. FET	D1,2,3,5 embryo	D1/2/3/5 embryo	A slow controlled-rate freezing procedure	Natural cycles/stimulated cycles	Preterm birth LBW
2008	Shih	Australia 1978–2005	Retro	IVF/ICSI/GIFT vs. FET	D2/D3 embryo	D2/D3 embryo	No specific description	Natural/artificial cycles	Preterm birth LBW Perinatal mortality Cesarean section
2010	Aflatoonian	Iran 2006–2008	Retro	IVF/ICSI vs. FET	D2/D3 embryo	D2/D3 embryo	Vitrification	Artificial cycles	Preterm birth LBW
2010	Pelkonen	Finland 1995–2006	Cohort study	IVF/ICSI vs. FET	D2/D3 embryo	D2/D3 embryo	Slow freezing	Natural/artificial cycles	Preterm birth LBW Still birth Perinatal mortality Cesarean section
2010	Pinborg	Denmark 1995–2007	Cohort study	IVF/ICSI vs. FET	D2/D3 embryo	D2/D3 embryo	Slow freezing	Not mentioned	Preterm birth LBW Still birth Perinatal mortality Cesarean section
2010	Wikland	Sweden 2006–2008	Cohort study	IVF/ICSI vs. FET	blastocysts	D2/D3 embryo/blastocysts	Vitrification/slow-freezing	Natural/artificial/stimulated cycles	Preterm birth LBW Perinatal mortality Cesarean section
2011	Henningsen	Denmark 1994–2006	Cohort study	IVF/ICSI vs. FET	D2 embryo	D2 embryo	Slow-freezing	Not mentioned	LBW
2012	Kalra	United States 2004–2006	Cohort study	IVF vs. FET	Not mentioned	Not mentioned	Not mentioned	Not mentioned	Preterm birth LBW
2012	Kato	Tokyo 2006–2008	Retro	IVF/ICSI vs. FET	Cleavage stage embryo/blastocyst	Cleavage stage embryo/ blastocyst	Vitrification	Natural/artificial cycles	Preterm birth LBW Still birth Perinatal mortality
2013	Wennerholm	Denmark −2007	Retro	IVF/ICSI vs. FET	D2 embryo	D2 embryo	Slow-freezing	Not mentioned	Preterm birth LBW Still birth Perinatal mortality Cesarean section
2015	Kemal	Antalya 2012–2012	Retro	IVF vs. FET	Blastocyst	Blastocyst	Vitrification	Artificial cycles	Still birth

*Abbreviation*: *Retro* retrospective

### Meta-analysis

**Fig. 1:**
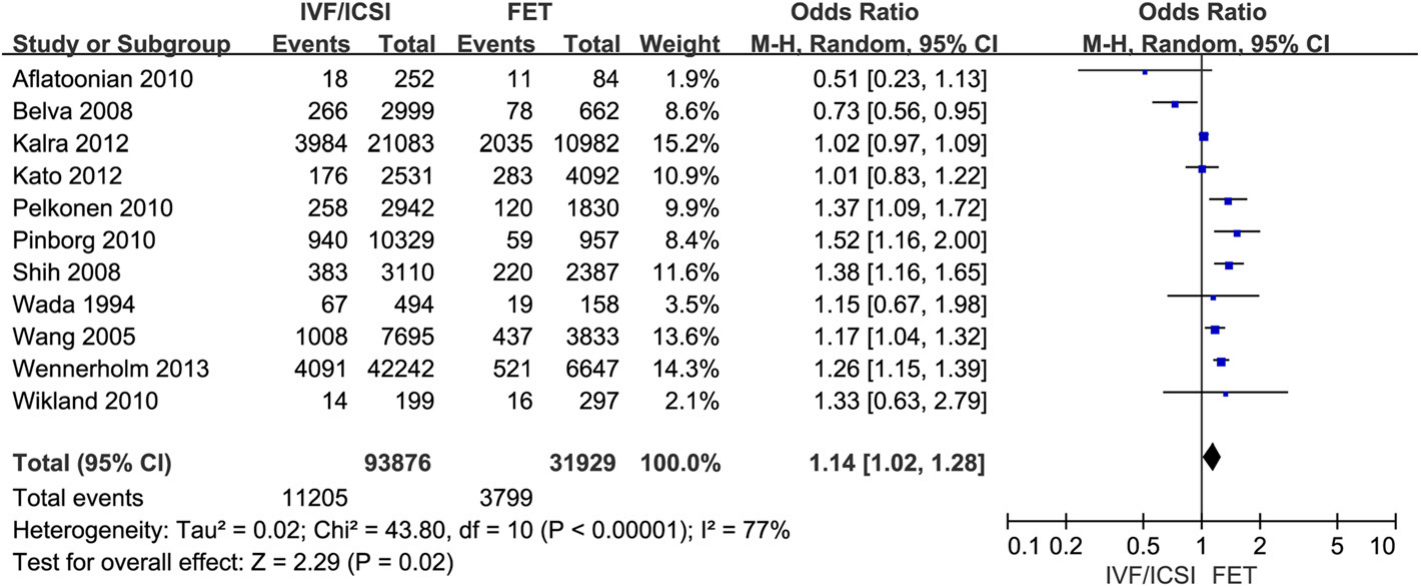
Forest plot showing the results of meta-analysis of studies comparing the preterm birth after IVF/ICSI vs. FET

**Fig. 2:**
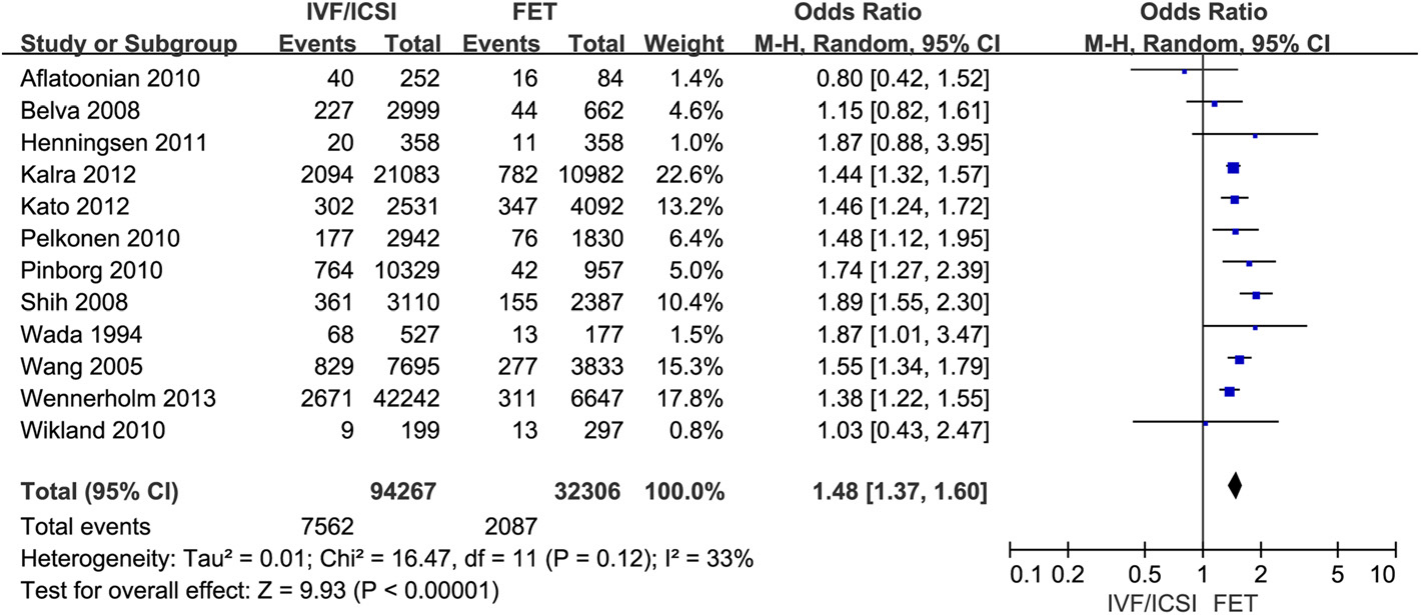
Forest plot showing the results of meta-analysis of studies comparing the LBW after IVF/ICSI vs. FET

Eleven studies were included in the present study comparing the preterm birth after IVF/ICSI vs. FET and 12 studies were enrolled to assess the LBW after IVF/ICSI vs. FET. We found a significantly decreased risk of preterm birth and low birth weight in singleton pregnancy resulting from FET compared with those after IVF/ICSI. In the assessment of preterm birth, the Q statistic *P*-value was below 0.1, indicating marked heterogeneity of the studies (l^2^ = 77 %, *P* < 0.01). The random effects model was implied and the combined OR was 1.14 (95 % CI, 1.02, 1.28; *P* = 0.02). Moderate statistical heterogeneity was seen in assessment of low birth weight, although there was no significance at *P* < 0.1 (l^2^ = 33 %, *P* = 0.12). The random effects model combined OR was 1.48 (95 % CI, 1.37, 1.60; *P* < 0.0001) (Figs. 1 and 2).

**Fig. 3:**
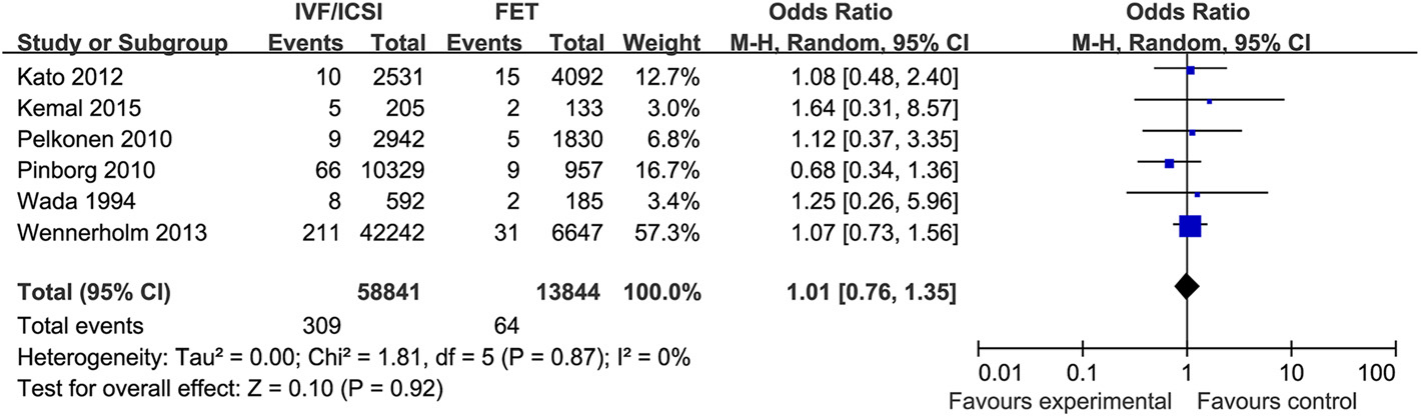
Forest plot showing the results of meta-analysis of studies comparing the still birth after IVF/ICSI vs. FET

**Fig. 4:**
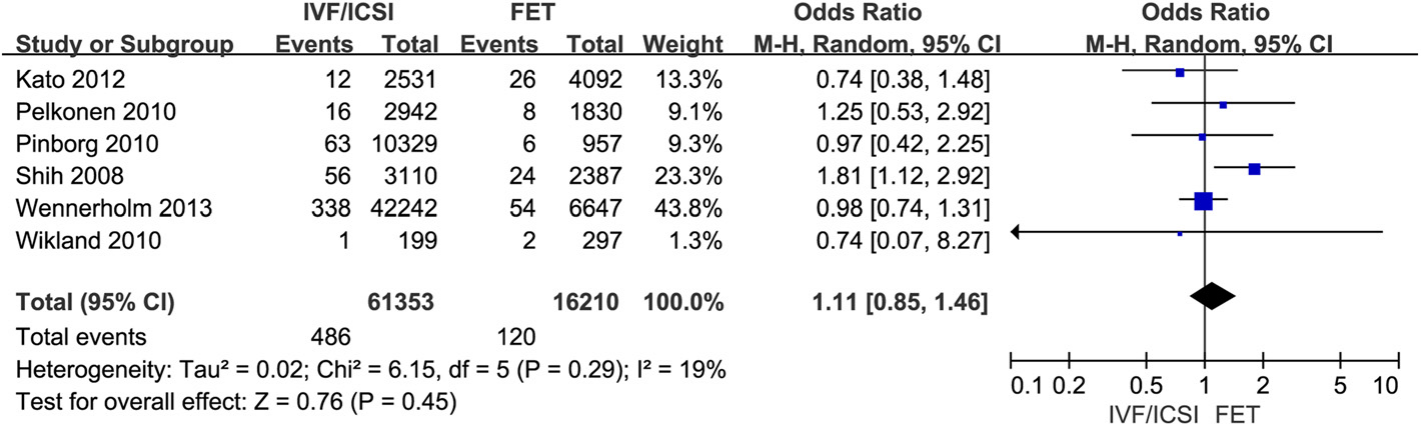
Forest plot showing the results of meta-analysis of studies comparing the perinatal mortality after IVF/ICSI vs. FET

Six studies compared the still birth and 5 studies compared perinatal mortality. The result of this study indicated that the risk of still birth and perinatal mortality was similar in singleton pregnancy after IVF/ICSI and FET cycles. The Q statistic *P*-values were 0.87 and 0.29, indicating zero and minimal heterogeneity among the studies, respectively (l^2^ = 0 %, *P* = 0.87; l^2^ = 19 %, *P* = 0.29). The fixed effects model was implied and the combined ORs were 1.01 (95 % CI, 0.76, 1.35; *P* = 0.92) and 1.11 (95 % CI, 0.85, 1.46; *P* = 0.45), respectively. (Figs. 3 and 4).

**Fig. 5:**
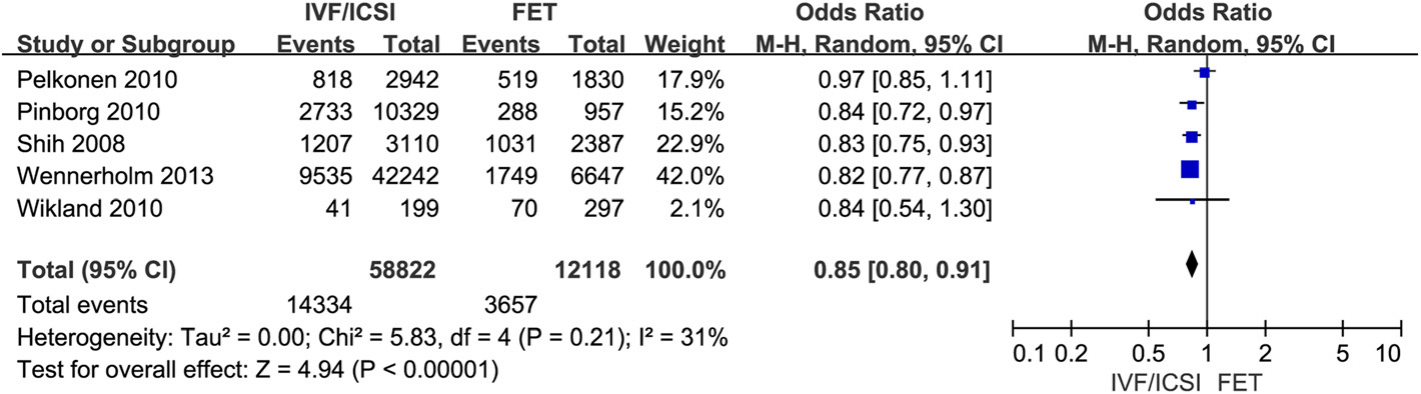
Forest plot showing the results of meta-analysis of studies comparing the cesarean section after IVF/ICSI vs. FET

At last, 5 studies were included to evaluate the cesarean section rate in singleton pregnancy after IVF/ICSI vs. FET. The result suggested that singleton pregnancy after IVF/ICSI was associated with decreased cesarean section rate compared with that of FET. There was minimal heterogeneity among studies as the Q statistic *P*-value was 0.21 and l^2^ was 31 %. The fixed effects model was used and the combined OR was 0.85 (95 % CI, 0.80, 0.91; *P* < 0.001) (Fig. 5).

The studies scored well on the Newcastle-Ottawa Quality Assessment Scale (not shown). The funnel plots of meta-analysis comparing the obstetric or perinatal outcomes after FET and IVF/ICIS did not find any publication bias due to its symmetrical shape. (Additional files 2, 3, 4, 5 and 6: Figures S2, S3, S4, S5 and S6).

## Discussions

So far, only one meta-analysis and systematic review [1] has compared the perinatal and obstetric outcomes in singleton pregnancy after IVF/ICSI and FET. To our knowledge, the present meta-analysis is the largest in regard to sample size with 94,472 IVF/ICSI cycles and 32,439 FET cycles. In the present meta-analysis, 11, 12, 6, 6, 5 studies were included to compare the risk of preterm birth, LBW, still birth, perinatal mortality, and cesarean section respectively. Our results indicated that singleton pregnancy after FET was at a decreased risk of LBW and preterm birth; however compared with IVF/ICSI cycles, singleton pregnancy after FET has a higher risk of cesarean section, which was in accordance with the former meta-analysis by Maheshwari et al. [1]. On the contrary, we found there was no significant difference in the risk of perinatal mortality. Additionally, the present study assessed the still birth for the first time, and the result demonstrated that there was no significant difference in the risk of still birth after the IVF/ICSI and the FET cycles.

The present meta-analysis suggested that the risks of preterm birth and LBW were decreased in singleton pregnancy subsequent to FET. Many other studies also found that there was lower risk of preterm birth, very preterm birth, low birth weight, small for gestational age, and perinatal mortality in FET pregnancies [16–19]. Another research did not find significant difference in the birth weights and preterm birth rates between singleton FET pregnancies and singleton spontaneous conceptions [20]. As preterm birth always accompanies with LBW, these two outcomes are related. A review by Evans et al. also concluded that FET was associated with reduced risk of ovarian hyperstimulation syndrome and improved outcomes for both mother and baby [21]. Why FET cycles have better outcomes compared with fresh ET is still not clear. The possible explanations may be as follows:

Firstly, FET involves in mini-stimulation or even no stimulation for ovarian. The endometrium was in the state of physiological condition, which may have a positive influence not only on the endometrial receptivity and early implantation but also on placentation and subsequent fetal growth [4, 21]. Two comparative studies also found that births from FET have a better perinatal outcome and a similar neonatal and birth outcome compared with fresh ET [1, 20], which confirmed the above assumptions.

Secondly, ovarian stimulation with a supraphysiologic hormone level in fresh embryo transfer cycles has negative effect on endometrial receptivity and embryos development, and results in the asynchronism between the embryo and endometrium which have detrimental effect on the development of embryo. Other studies suggested that ovarian hyper-stimulation during fresh cycles change angiogenesis of endometrium and embryo imbed [22–24].

Thirdly, the process of FET involving embryo cryopreservation and embryo thawing would weed out poor quality embryos, and permit top quality embryos to survive, leading to a better clinical outcome [25]. In fresh IVF/ICSI cycles, embryos in normal morphology with less development potential are more likely transferred.

In the present study, we also revealed that there was similar risk of the perinatal mortality and still birth in singleton pregnancy between IVF/ICSI and FET. Whereas, the previous study done by Maheshwari et al. [1] believed that a lower risk of perinatal mortality in singleton pregnancy was associated with FET. The difference in results may be because of the difference between studies included. Besides, the rate of cesarean section in pregnancies subsequent to FET was higher than that after IVF/ICSI. The possible reason may be that women undergoing FET were more likely to have previous cesarean sections compared with women undergoing fresh embryo transfer. Besides, pregnant women after FET may have attempted many times and conceived finally, and they considered the cesarean as a safer way to deliver and preferred to choose cesarean section.

The limitations of the present study embody the integral defect of studies included: variation in design, exclusion & inclusion criteria, definition of outcomes, methodological differences, small number of study subjects, imprecise information on drug exposures, and lack of adjustment for meaningful confounders. However, it was impossible for us to adjust for some confounders due to lack of individual patient data.

Notwithstanding these limitations, the present meta-analysis and systematic review provides a valuable summary of the results of published studies. From what we have discussed above, singleton pregnancy after FET has a lower risk of preterm birth, LBW than that after fresh IVF/ICSI-ET cycles, and has a similar risk of perinatal mortality and still birth with that after IVF/ICSI. With the improvement of cryopreservation facilities and techniques, elective cryopreservation for later use may be recommended. But the clinical and cost effectiveness of the elective cryopreservation as well as acceptability of infertile couple should be evaluated before this strategy applied into clinical practice.

## Conclusions

Singleton pregnancy after FET seems to have a better perinatal outcome compared with that after IVF/ICSI. Considering limitation of this present study, further cohort studies which adjust for a variety of meaningful confounders are needed in order to draw sound conclusions.

## Additional files

Additional file 1: Figure S1. Flow chart showing study selection process (TIF 638 kb) Additional file 2: Figure S2. Funnel plot of analysis for the comparation of preterm birth in singleton pregnancy after IVF/ICSI vs. FET, showing the results of Eggers to assess publication bias (TIF 275 kb) Additional file 3: Figure S3. Funnel plot of analysis for the comparation of LBW in singleton pregnancy after IVF/ICSI vs. FET, showing the results of Eggers to assess publication bias (TIF 276 kb) Additional file 4: Figure S4. Funnel plot of analysis for the comparation of still birth in singleton pregnancy after IVF/ICSI vs. FET, showing the results of Eggers to assess publication bias (TIF 275 kb) Additional file 5: Figure S5. Funnel plot of analysis for the comparation of perinatal mortality in singleton pregnancy after IVF/ICSI vs. FET, showing the results of Eggers to assess publication bias (TIF 267 kb) Additional file 6: Figure S6. Funnel plot of analysis for the comparation of cesarean section in singleton pregnancy after IVF/ICSI vs. FET, showing the results of Eggers to assess publication bias (TIF 269 kb)

## Abbreviations

COS: Controlled ovarian stimulation
FET: Frozen embryo transfer
ICSI: Intracytoplasmic sperm injection
IVF: In vitro fertilization
OHSS: Ovarian hyperstimulation syndrome
