# Supplementary figures and images for: Which one has a better obstetric and perinatal outcome in singleton pregnancy, IVF/ICSI or FET?: a systematic review and meta-analysis
Source: Reprod Biol Endocrinol. 2016 Aug 30;14(1):51. doi: 10.1186/s12958-016-0188-3 (PMC5006501; doi:10.1186/s12958-016-0188-3)

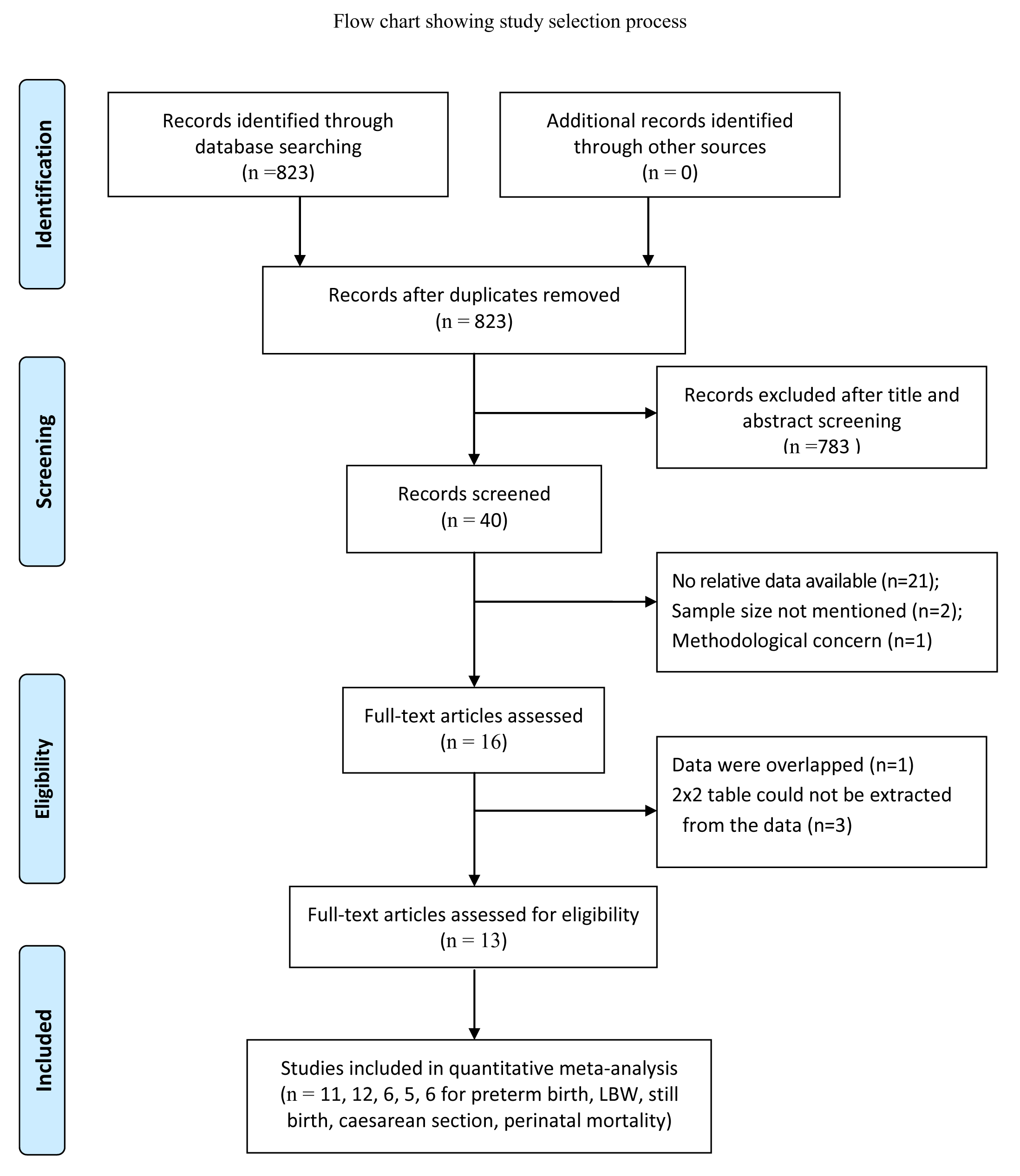

Supplement: Additional file 1: Figure S1. — Flow chart showing study selection process (TIF 638 kb) [file 12958_2016_188_MOESM1_ESM.tif]

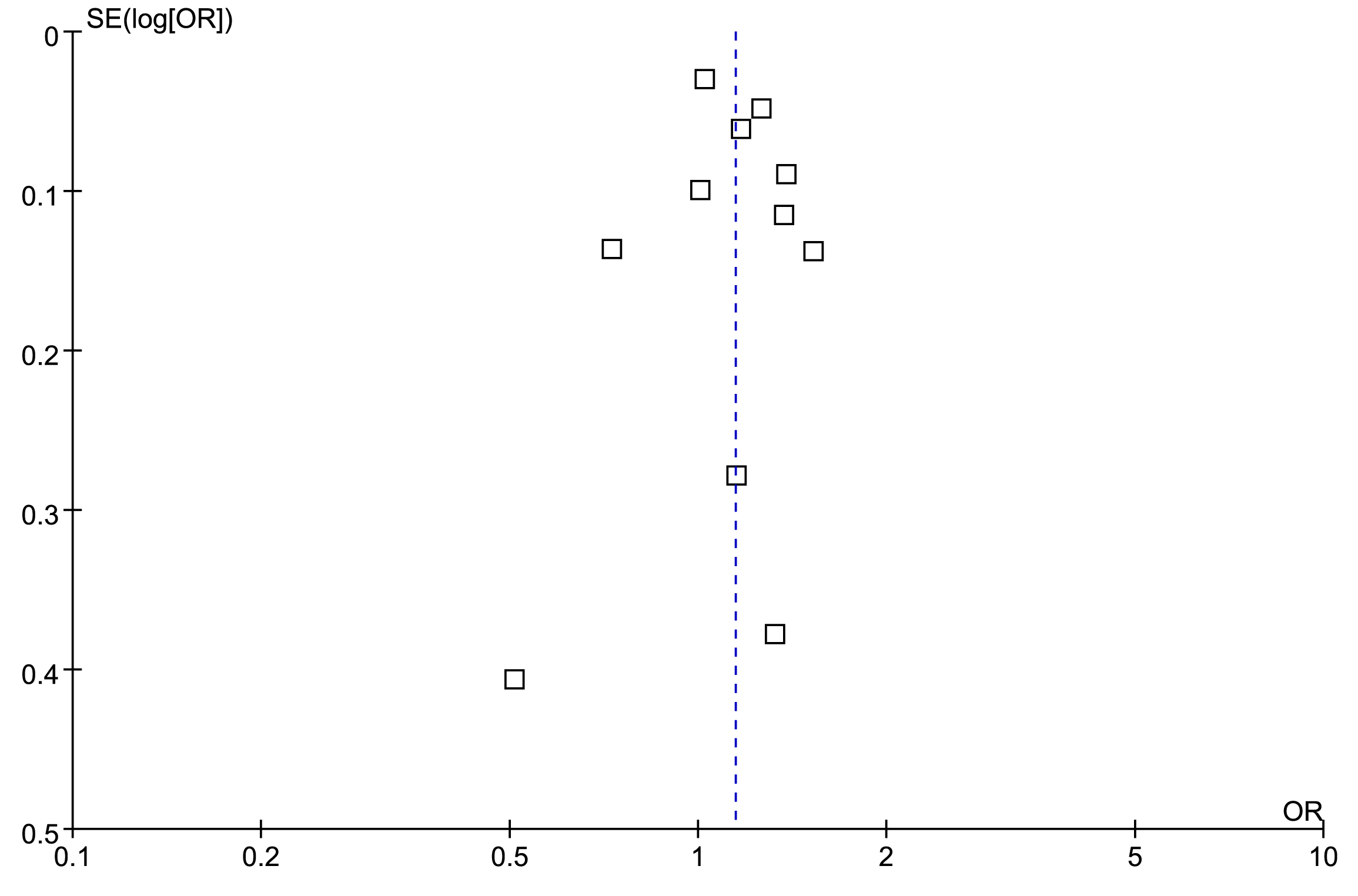

Supplement: Additional file 2: Figure S2. — Funnel plot of analysis for the comparation of preterm birth in singleton pregnancy after IVF/ICSI vs. FET, showing the results of Eggers to assess publication bias (TIF 275 kb) [file 12958_2016_188_MOESM2_ESM.tif]

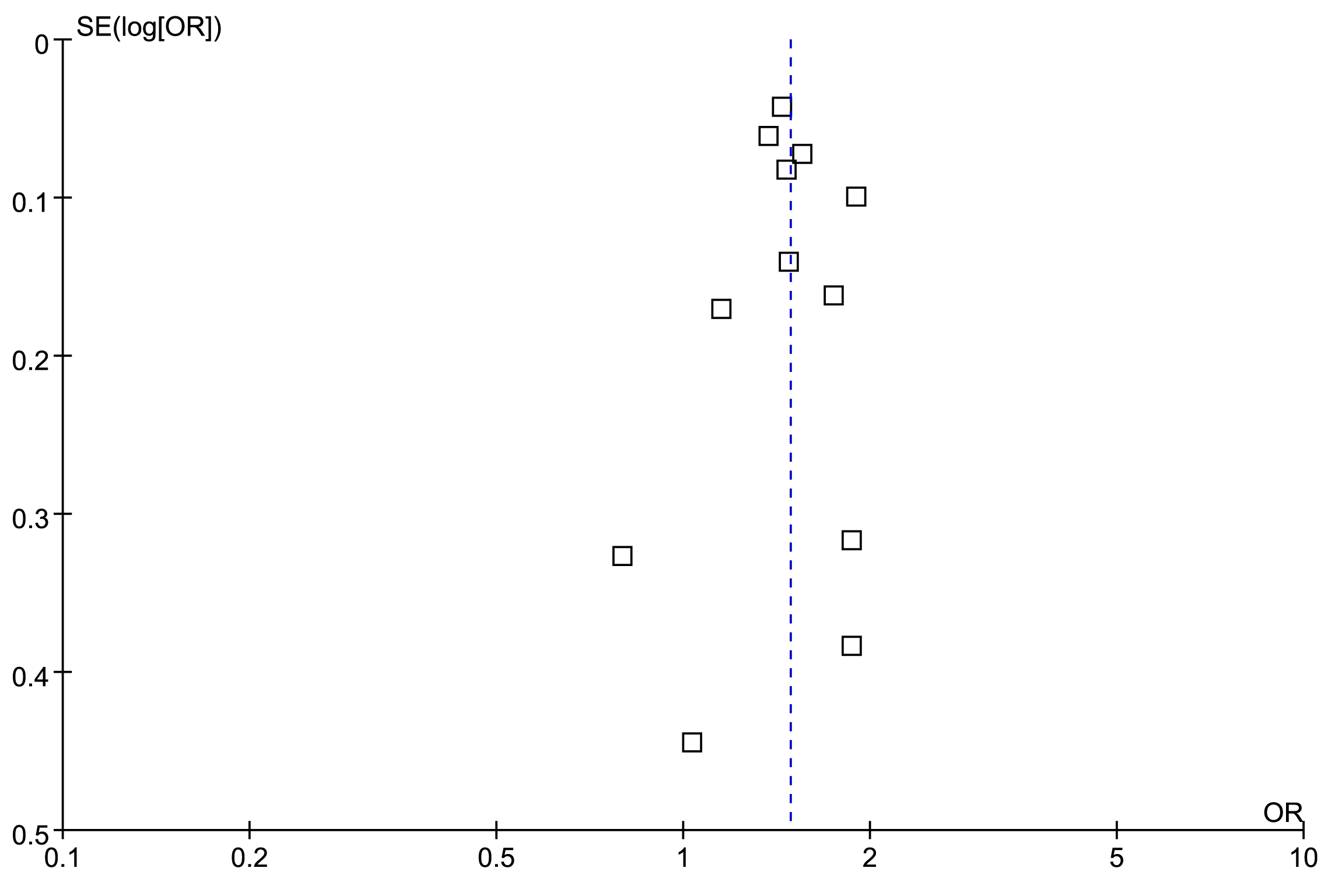

Supplement: Additional file 3: Figure S3. — Funnel plot of analysis for the comparation of LBW in singleton pregnancy after IVF/ICSI vs. FET, showing the results of Eggers to assess publication bias (TIF 276 kb) [file 12958_2016_188_MOESM3_ESM.tif]

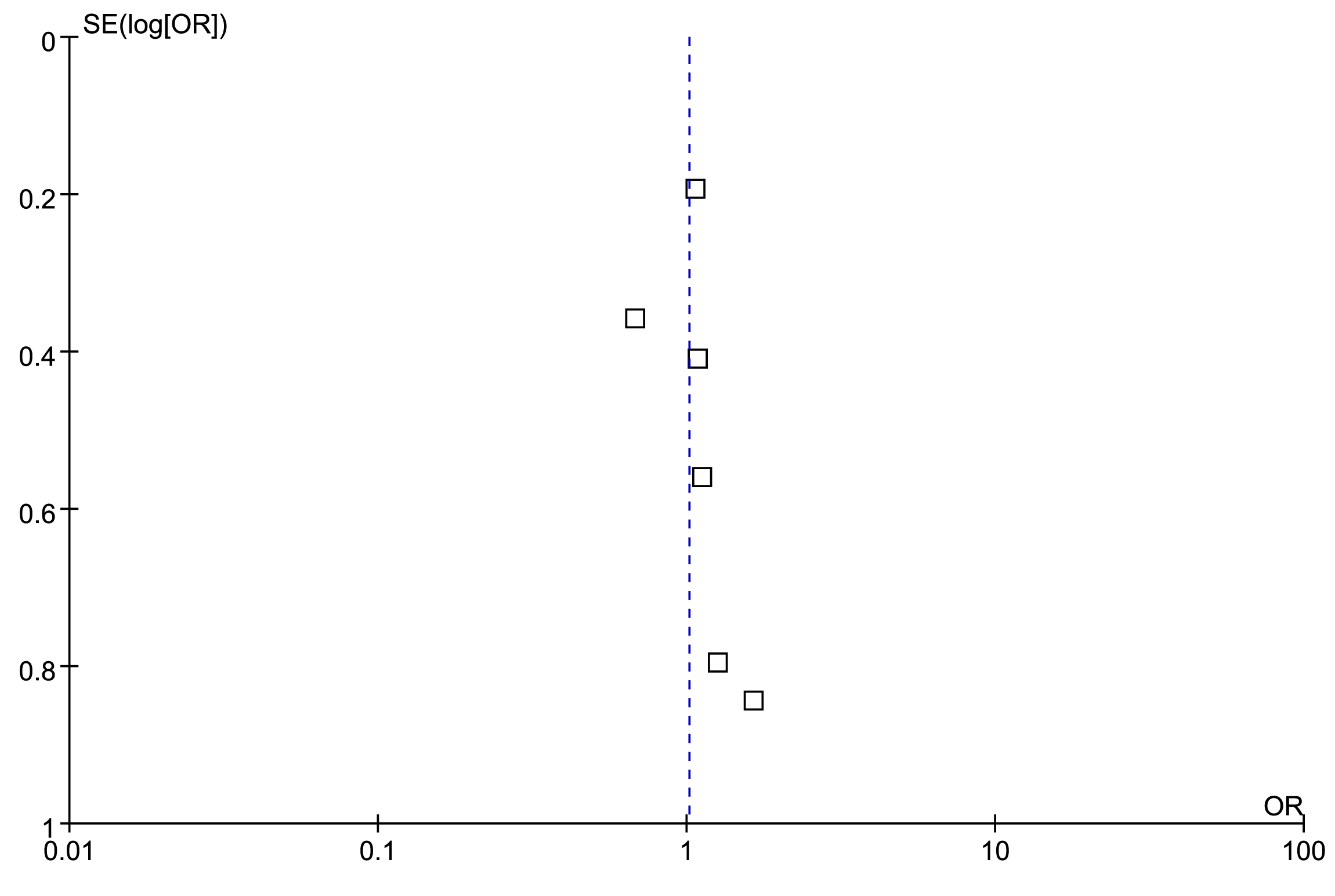

Supplement: Additional file 4: Figure S4. — Funnel plot of analysis for the comparation of still birth in singleton pregnancy after IVF/ICSI vs. FET, showing the results of Eggers to assess publication bias (TIF 275 kb) [file 12958_2016_188_MOESM4_ESM.tif]

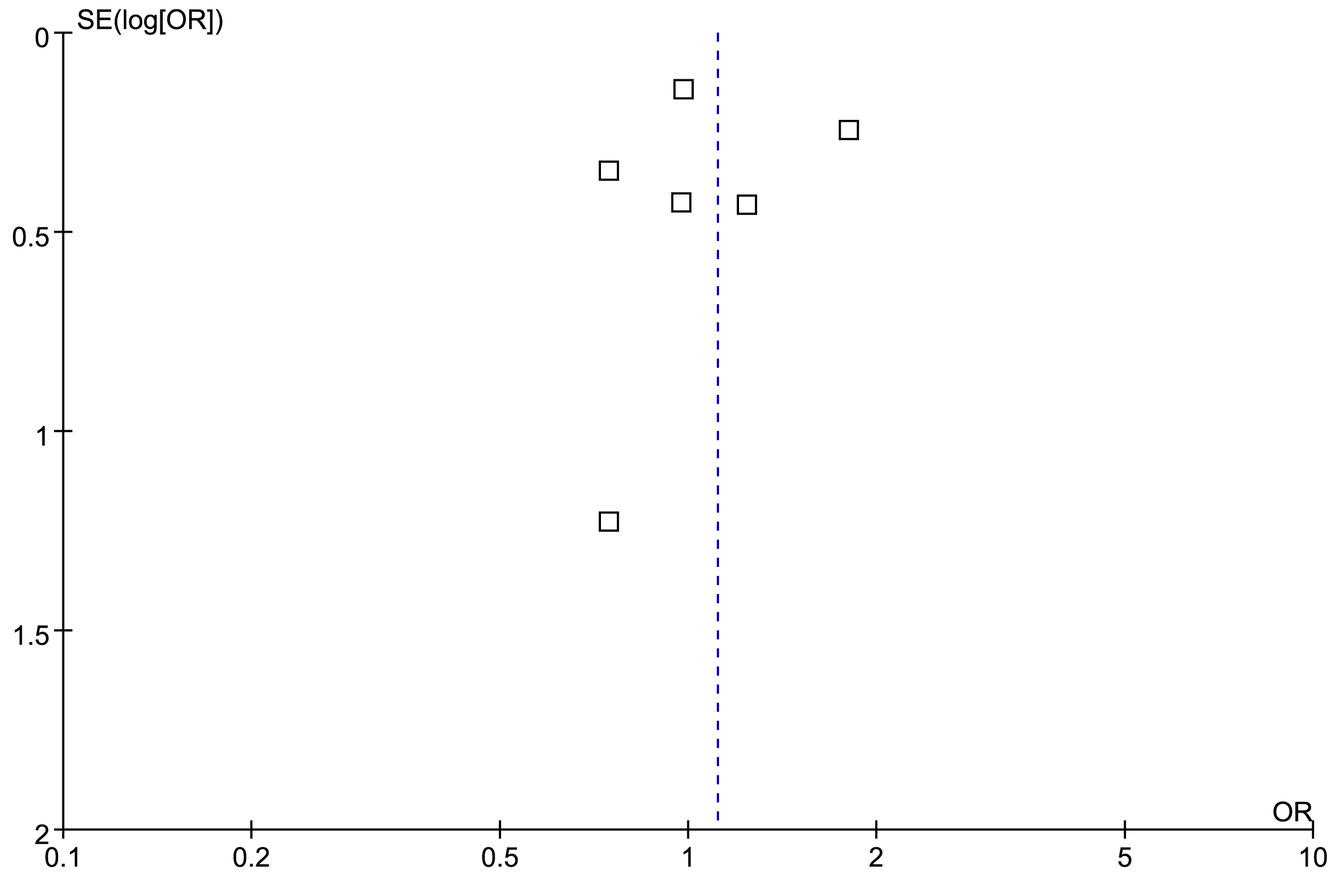

Supplement: Additional file 5: Figure S5. — Funnel plot of analysis for the comparation of perinatal mortality in singleton pregnancy after IVF/ICSI vs. FET, showing the results of Eggers to assess publication bias (TIF 267 kb) [file 12958_2016_188_MOESM5_ESM.tif]

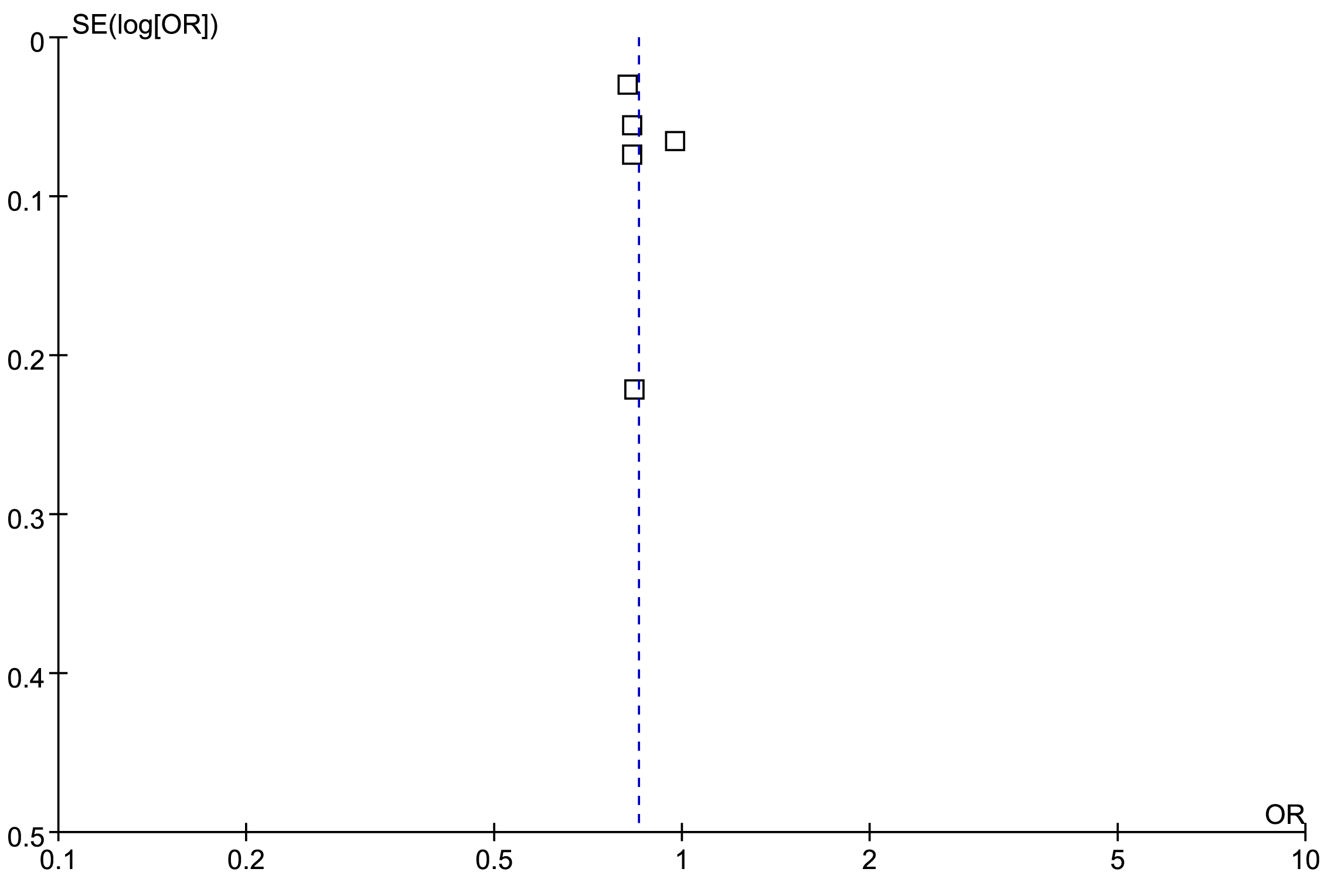

Supplement: Additional file 6: Figure S6. — Funnel plot of analysis for the comparation of cesarean section in singleton pregnancy after IVF/ICSI vs. FET, showing the results of Eggers to assess publication bias (TIF 269 kb) [file 12958_2016_188_MOESM6_ESM.tif]
